# Ethyl 6-methyl-2-oxo-4-phenyl-1,2,3,4-tetra­hydro­pyrimidine-5-carboxyl­ate

**DOI:** 10.1107/S1600536808025610

**Published:** 2008-08-13

**Authors:** M. Nizam Mohideen, A. Rasheeth, C. A. M. A. Huq, S. Syed Nizar

**Affiliations:** a Department of Physics, The New College (Autonomous), Chennai 600 014, India; b Department of Chemistry, The New College (Autonomous), Chennai 600 014, India

## Abstract

The title compound, C_14_H_16_N_2_O_3_, belongs to a group of esters of 2-oxo- and 1,2,3,4-tetra­hydro­pyrimidine-5-carboxylic acids, which exhibit a wide spectrum of biological activities. The dihydro­pyrimidine ring adopts a screw-boat conformation. The crystal packing is stabilized by strong N—H⋯O and weak C—H⋯O inter­molecular hydrogen bonds. An intra­molecular C—H⋯O hydrogen bond is also present.

## Related literature

For related literature, see: Atwal *et al.* (1991 ▶); Broughton *et al.* (1975 ▶); Cremer & Pople (1975 ▶); Kappe *et al.* (1997 ▶); Li *et al.* (2005 ▶); Nardelli (1983 ▶); Overman *et al.* (1995 ▶).
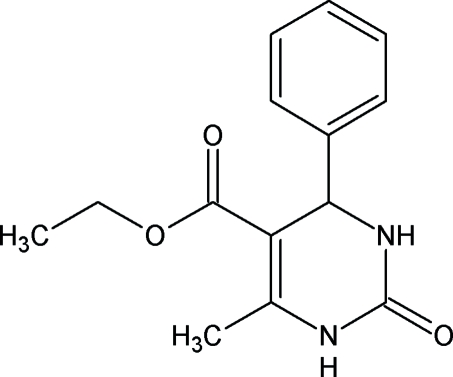


## Experimental

### 

#### Crystal data

C_14_H_16_N_2_O_3_
*M* *_r_* = 260.29
Triclinic, 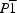

*a* = 7.5495 (2) Å
*b* = 8.9797 (3) Å
*c* = 11.0812 (3) Å
α = 107.843 (2)°
β = 108.603 (1)°
γ = 98.244 (2)°
*V* = 653.07 (4) Å^3^
*Z* = 2
Mo *K*α radiation
μ = 0.09 mm^−1^
*T* = 293 (2) K
0.3 × 0.2 × 0.2 mm

#### Data collection

Bruker APEXII CCD area-detector diffractometer
Absorption correction: none
16135 measured reflections
3710 independent reflections
2972 reflections with *I* > 2σ(*I*)
*R*_int_ = 0.023

#### Refinement

*R*[*F* ^2^ > 2σ(*F* ^2^)] = 0.047
*wR*(*F* ^2^) = 0.159
*S* = 1.00
3710 reflections
174 parameters
H-atom parameters constrained
Δρ_max_ = 0.34 e Å^−3^
Δρ_min_ = −0.33 e Å^−3^

### 

Data collection: *APEX2* (Bruker, 2004 ▶); cell refinement: *APEX2* and *SAINT* (Bruker, 2004 ▶); data reduction: *SAINT*; program(s) used to solve structure: *SHELXS97* (Sheldrick, 2008 ▶); program(s) used to refine structure: *SHELXL97* (Sheldrick, 2008 ▶); molecular graphics: *ORTEP-3 for Windows* (Farrugia, 1997 ▶); software used to prepare material for publication: *SHELXL97* and *PLATON* (Spek, 2003 ▶).

## Supplementary Material

Crystal structure: contains datablocks global, I. DOI: 10.1107/S1600536808025610/bt2758sup1.cif

Structure factors: contains datablocks I. DOI: 10.1107/S1600536808025610/bt2758Isup2.hkl

Additional supplementary materials:  crystallographic information; 3D view; checkCIF report

## Figures and Tables

**Table 1:** Hydrogen-bond geometry (Å, °)

*D*—H⋯*A*	*D*—H	H⋯*A*	*D*⋯*A*	*D*—H⋯*A*
N1—H1⋯O2^i^	0.86	2.37	3.1773 (13)	156
N2—H2⋯O1^ii^	0.86	2.00	2.8568 (13)	178
C11—H11*A*⋯O1^iii^	0.96	2.58	3.1785 (16)	121
C11—H11*C*⋯O2	0.96	2.44	2.8379 (17)	105

Symmetry codes: (i) 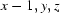; (ii) 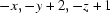; (iii) 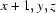.

**Fig. 1.**
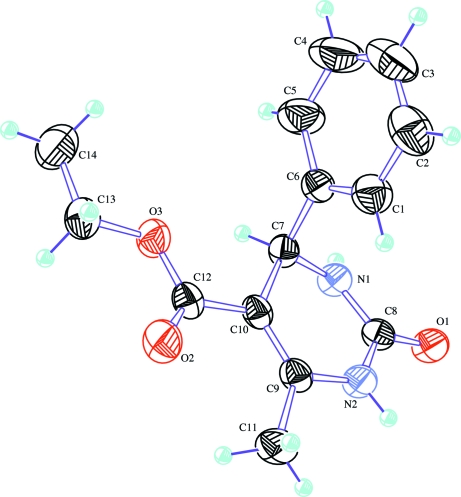
The molecular configuration and atom-numbering scheme for (I). Displacement ellipsoids are drawn at the 50% probability level.

## supplementary crystallographic information

### Comment

The title compound belongs to the group of esters of 2-oxo and -1,2,3,4-tetrahydropyrimidine-5-carboxylic acids, which are known as `Biginelli compounds' (Kappe *et al.*,  1997). It has been suggested that the substituent effect may be attributable to intramolecular hydrogen bonding between the alkoxy oxygen and the proton of the pyrimidine ring NH group (Broughton *et al.*, 1975). Several marine alkaloids having the dihydropyrimidine core unit have been found to show interesting biological activities, such as antiviral, antibacterial and anti-inflammatory (Overman *et al.*, 1995). Many functionalized derivatives are used as calcium channel blockers and antihypertensive agents (Atwal *et al.*, 1991). Against this background and in order to obtain detailed information on its molecular conformation, the structure of the title compound has been determined and the results are presented here.

The bond lengths and angles are comparable with the similar structure reported in the literature (Li *et al.*, 2005). The six membered ring (atoms N1, N2, C7, C8, C9, C10) of the dihydropyrimidine group adopts a screw boat conformation; the puckering parameters are q_2_ = 0.257 (1) Å and φ = 211.7 (2)° (Cremer & Pople, 1975), and the lowest displacement asymmetry parameters Δ_S_(N1) is 14.4 (1)° (Nardelli, 1983), with atom O1 deviating by -0.116 (1) Å from the least squares plane of the ring.

The dihedral angle between the pyrimidine and benzene rings is 86.5 (1)°, close to the value of 82.8 (6)° found in ethyl 4-(4-hydroxyphenyl)-6-methyl-2-oxo-1,2,3,4-tetrahydropyrimidine-5- carboxylate.

The crystal packing is stabilized by strong N—H···O and C—H···O intermolecular hydrogen bonds (Table 1).

### Experimental

A mixture of benzaldehyde (0.106 g, 1 mmol), ethyl acetoacetate (0.130 g, 1 mmol) and urea (0.070 g, 1.17 mmol) was ground with four drops of *ortho* phosphoric acid for about 30 minutes. The reaction mixture was cooled for 15 minutes and poured into a beaker containing 50 ml of cold water. The precipitate obtained was filtered, washed with water and ethanol to get white solid. Single crystals of the title compound suitable for X-ray diffraction were obtained by slow evaporation of a solution in ethanol (0.26 g, 92% yield; mp 203–204).

### Refinement

All H atoms were positioned geometrically and allowed to ride on their parent C atoms, with C—H distances fixed in the range 0.93–0.98 Å and N—H distance of 0.86 Å, with *U*_iso_(H) = 1.5*U*_eq_(C) for methyl H atoms and *U*_iso_(H) = 1.2*U*_eq_(C, N) for other H atoms.

### Crystal data

**Table:**

C_14_H_16_N_2_O_3_	*Z* = 2
*M**_r_* = 260.29	*F*_000_ = 276
Triclinic, *P*1	*D*_x_ = 1.324 Mg m^−^^3^
Hall symbol: -P 1	Mo *K*α radiation λ = 0.71073 Å
*a* = 7.5495 (2) Å	Cell parameters from 5321 reflections
*b* = 8.9797 (3) Å	θ = 2.5–24.1º
*c* = 11.0812 (3) Å	µ = 0.09 mm^−^^1^
α = 107.843 (2)º	*T* = 293 (2) K
β = 108.603 (1)º	Needle, colourless
γ = 98.244 (2)º	0.3 × 0.2 × 0.2 mm
*V* = 653.07 (4) Å^3^	

### Data collection

**Table:**

Bruker APEXII CCD area-detector diffractometer	2972 reflections with *I* > 2σ(*I*)
Radiation source: fine-focus sealed tube	*R*_int_ = 0.023
Monochromator: graphite	θ_max_ = 29.7º
*T* = 293(2) K	θ_min_ = 2.5º
ω and φ scans	*h* = −9→10
Absorption correction: None	*k* = −12→12
16135 measured reflections	*l* = −15→15
3710 independent reflections	

### Refinement

**Table:**

Refinement on *F*^2^	Secondary atom site location: difference Fourier map
Least-squares matrix: full	Hydrogen site location: inferred from neighbouring sites
*R*[*F*^2^ > 2σ(*F*^2^)] = 0.047	H-atom parameters constrained
*wR*(*F*^2^) = 0.159	*w* = 1/[σ^2^(*F*_o_^2^) + (0.0959*P*)^2^ + 0.106*P*] where *P* = (*F*_o_^2^ + 2*F*_c_^2^)/3
*S* = 1.00	(Δ/σ)_max_ < 0.001
3710 reflections	Δρ_max_ = 0.34 e Å^−^^3^
174 parameters	Δρ_min_ = −0.33 e Å^−^^3^
Primary atom site location: structure-invariant direct methods	Extinction correction: none

### Special details

**Table:**

Geometry. All e.s.d.'s (except the e.s.d. in the dihedral angle between two l.s. planes) are estimated using the full covariance matrix. The cell e.s.d.'s are taken into account individually in the estimation of e.s.d.'s in distances, angles and torsion angles; correlations between e.s.d.'s in cell parameters are only used when they are defined by crystal symmetry. An approximate (isotropic) treatment of cell e.s.d.'s is used for estimating e.s.d.'s involving l.s. planes.
Refinement. Refinement of *F*^2^ against ALL reflections. The weighted *R*-factor *wR* and goodness of fit *S* are based on *F*^2^, conventional *R*-factors *R* are based on *F*, with *F* set to zero for negative *F*^2^. The threshold expression of *F*^2^ > σ(*F*^2^) is used only for calculating *R*-factors(gt) *etc*. and is not relevant to the choice of reflections for refinement. *R*-factors based on *F*^2^ are statistically about twice as large as those based on *F*, and *R*- factors based on ALL data will be even larger.

### Fractional atomic coordinates and isotropic or equivalent isotropic displacement parameters (Å^2^)

**Table:**

	*x*	*y*	*z*	*U*_iso_*/*U*_eq_	
O1	−0.18468 (12)	0.90274 (12)	0.54481 (10)	0.0472 (2)	
O2	0.51966 (14)	0.59340 (12)	0.67258 (11)	0.0546 (3)	
O3	0.30937 (13)	0.47104 (11)	0.73822 (10)	0.0473 (2)	
N1	−0.09885 (13)	0.70875 (12)	0.62494 (10)	0.0389 (2)	
H1	−0.2165	0.6651	0.6107	0.047*	
N2	0.10294 (14)	0.85056 (13)	0.55737 (11)	0.0413 (2)	
H2	0.1288	0.9264	0.5285	0.050*	
C1	0.2277 (2)	0.89996 (17)	0.91862 (15)	0.0542 (3)	
H1A	0.2782	0.9495	0.8699	0.065*	
C2	0.2660 (3)	0.9866 (2)	1.05616 (17)	0.0711 (5)	
H2A	0.3398	1.0942	1.0982	0.085*	
C3	0.1961 (3)	0.9148 (3)	1.12917 (17)	0.0757 (5)	
H3	0.2226	0.9726	1.2212	0.091*	
C4	0.0872 (4)	0.7575 (3)	1.0669 (2)	0.0860 (6)	
H4	0.0400	0.7079	1.1170	0.103*	
C5	0.0461 (3)	0.6709 (2)	0.93002 (18)	0.0663 (4)	
H5	−0.0292	0.5638	0.8885	0.080*	
C6	0.11595 (16)	0.74226 (14)	0.85451 (12)	0.0380 (2)	
C7	0.05617 (15)	0.65119 (13)	0.70151 (12)	0.0351 (2)	
H7	0.0032	0.5360	0.6808	0.042*	
C8	−0.06779 (16)	0.82505 (14)	0.57613 (12)	0.0363 (2)	
C9	0.23610 (16)	0.76219 (13)	0.58195 (12)	0.0371 (2)	
C10	0.22084 (15)	0.66550 (13)	0.65175 (11)	0.0353 (2)	
C11	0.3835 (2)	0.7861 (2)	0.52121 (17)	0.0556 (4)	
H11A	0.5017	0.8612	0.5918	0.083*	
H11B	0.3354	0.8286	0.4511	0.083*	
H11C	0.4080	0.6839	0.4817	0.083*	
C12	0.36538 (16)	0.57600 (13)	0.68571 (12)	0.0387 (2)	
C13	0.4443 (2)	0.37945 (18)	0.77957 (16)	0.0533 (3)	
H13A	0.5686	0.4526	0.8461	0.064*	
H13B	0.4638	0.3116	0.7006	0.064*	
C14	0.3605 (3)	0.2774 (2)	0.8412 (2)	0.0736 (5)	
H14A	0.3373	0.3455	0.9171	0.110*	
H14B	0.4495	0.2185	0.8729	0.110*	
H14C	0.2403	0.2023	0.7733	0.110*	

### Atomic displacement parameters (Å^2^)

**Table:**

	*U*^11^	*U*^22^	*U*^33^	*U*^12^	*U*^13^	*U*^23^
O1	0.0364 (4)	0.0620 (5)	0.0619 (5)	0.0227 (4)	0.0271 (4)	0.0348 (5)
O2	0.0412 (5)	0.0638 (6)	0.0791 (7)	0.0260 (4)	0.0348 (5)	0.0359 (5)
O3	0.0454 (5)	0.0496 (5)	0.0621 (6)	0.0229 (4)	0.0290 (4)	0.0276 (4)
N1	0.0265 (4)	0.0462 (5)	0.0487 (5)	0.0090 (4)	0.0175 (4)	0.0211 (4)
N2	0.0337 (5)	0.0488 (5)	0.0569 (6)	0.0163 (4)	0.0268 (4)	0.0281 (5)
C1	0.0591 (8)	0.0482 (7)	0.0481 (7)	0.0069 (6)	0.0199 (6)	0.0127 (6)
C2	0.0693 (11)	0.0669 (9)	0.0531 (8)	0.0156 (8)	0.0146 (7)	0.0016 (7)
C3	0.0706 (11)	0.1122 (15)	0.0470 (8)	0.0445 (11)	0.0283 (8)	0.0187 (9)
C4	0.1003 (15)	0.1211 (18)	0.0684 (11)	0.0388 (14)	0.0585 (11)	0.0462 (12)
C5	0.0736 (10)	0.0732 (10)	0.0667 (9)	0.0123 (8)	0.0443 (8)	0.0307 (8)
C6	0.0327 (5)	0.0453 (6)	0.0446 (6)	0.0160 (4)	0.0208 (4)	0.0195 (5)
C7	0.0300 (5)	0.0336 (5)	0.0453 (6)	0.0088 (4)	0.0185 (4)	0.0152 (4)
C8	0.0290 (5)	0.0425 (5)	0.0397 (5)	0.0100 (4)	0.0162 (4)	0.0150 (4)
C9	0.0304 (5)	0.0407 (5)	0.0434 (6)	0.0112 (4)	0.0194 (4)	0.0138 (4)
C10	0.0306 (5)	0.0357 (5)	0.0410 (5)	0.0109 (4)	0.0182 (4)	0.0109 (4)
C11	0.0466 (7)	0.0753 (9)	0.0742 (9)	0.0281 (7)	0.0421 (7)	0.0416 (8)
C12	0.0353 (5)	0.0382 (5)	0.0436 (6)	0.0133 (4)	0.0184 (4)	0.0116 (4)
C13	0.0531 (8)	0.0576 (7)	0.0645 (8)	0.0292 (6)	0.0289 (7)	0.0302 (7)
C14	0.0817 (12)	0.0773 (11)	0.0928 (13)	0.0373 (9)	0.0446 (10)	0.0539 (10)

### Geometric parameters (Å, °)

**Table:**

O1—C8	1.2310 (14)	C5—C6	1.3793 (18)
O2—C12	1.2133 (14)	C5—H5	0.9300
O3—C12	1.3359 (15)	C6—C7	1.5176 (16)
O3—C13	1.4481 (15)	C7—C10	1.5168 (14)
N1—C8	1.3398 (15)	C7—H7	0.9800
N1—C7	1.4716 (15)	C9—C10	1.3436 (16)
N1—H1	0.8600	C9—C11	1.4950 (15)
N2—C8	1.3684 (14)	C10—C12	1.4673 (15)
N2—C9	1.3788 (14)	C11—H11A	0.9600
N2—H2	0.8600	C11—H11B	0.9600
C1—C6	1.3712 (18)	C11—H11C	0.9600
C1—C2	1.393 (2)	C12—O2	1.2133 (14)
C1—H1A	0.9300	C13—C14	1.483 (2)
C2—C3	1.357 (3)	C13—H13A	0.9700
C2—H2A	0.9300	C13—H13B	0.9700
C3—C4	1.362 (3)	C14—H14A	0.9600
C3—H3	0.9300	C14—H14B	0.9600
C4—C5	1.382 (3)	C14—H14C	0.9600
C4—H4	0.9300		
			
C12—O3—C13	116.09 (10)	O1—C8—N2	120.40 (10)
C8—N1—C7	123.94 (9)	N1—C8—N2	115.72 (10)
C8—N1—H1	118.0	C10—C9—N2	119.72 (10)
C7—N1—H1	118.0	C10—C9—C11	127.49 (11)
C8—N2—C9	124.03 (10)	N2—C9—C11	112.77 (10)
C8—N2—H2	118.0	C9—C10—C12	121.18 (10)
C9—N2—H2	118.0	C9—C10—C7	120.31 (10)
C6—C1—C2	120.50 (14)	C12—C10—C7	118.47 (10)
C6—C1—H1A	119.8	C9—C11—H11A	109.5
C2—C1—H1A	119.8	C9—C11—H11B	109.5
C3—C2—C1	120.33 (17)	H11A—C11—H11B	109.5
C3—C2—H2A	119.8	C9—C11—H11C	109.5
C1—C2—H2A	119.8	H11A—C11—H11C	109.5
C2—C3—C4	119.62 (15)	H11B—C11—H11C	109.5
C2—C3—H3	120.2	O2—C12—O3	122.18 (11)
C4—C3—H3	120.2	O2—C12—O3	122.18 (11)
C3—C4—C5	120.60 (17)	O2—C12—C10	126.44 (11)
C3—C4—H4	119.7	O2—C12—C10	126.44 (11)
C5—C4—H4	119.7	O3—C12—C10	111.36 (9)
C6—C5—C4	120.44 (17)	O3—C13—C14	107.69 (12)
C6—C5—H5	119.8	O3—C13—H13A	110.2
C4—C5—H5	119.8	C14—C13—H13A	110.2
C1—C6—C5	118.50 (13)	O3—C13—H13B	110.2
C1—C6—C7	121.28 (11)	C14—C13—H13B	110.2
C5—C6—C7	120.04 (12)	H13A—C13—H13B	108.5
N1—C7—C10	109.36 (9)	C13—C14—H14A	109.5
N1—C7—C6	109.37 (9)	C13—C14—H14B	109.5
C10—C7—C6	114.22 (9)	H14A—C14—H14B	109.5
N1—C7—H7	107.9	C13—C14—H14C	109.5
C10—C7—H7	107.9	H14A—C14—H14C	109.5
C6—C7—H7	107.9	H14B—C14—H14C	109.5
O1—C8—N1	123.84 (10)		
			
C6—C1—C2—C3	1.3 (3)	C8—N2—C9—C11	165.78 (12)
C1—C2—C3—C4	−0.4 (3)	N2—C9—C10—C12	−176.93 (10)
C2—C3—C4—C5	−0.4 (3)	C11—C9—C10—C12	4.52 (19)
C3—C4—C5—C6	0.4 (3)	N2—C9—C10—C7	0.98 (17)
C2—C1—C6—C5	−1.3 (2)	C11—C9—C10—C7	−177.57 (12)
C2—C1—C6—C7	173.94 (13)	N1—C7—C10—C9	18.31 (14)
C4—C5—C6—C1	0.4 (3)	C6—C7—C10—C9	−104.61 (12)
C4—C5—C6—C7	−174.83 (16)	N1—C7—C10—C12	−163.72 (9)
C8—N1—C7—C10	−31.02 (15)	C6—C7—C10—C12	73.35 (12)
C8—N1—C7—C6	94.75 (12)	C13—O3—C12—O2	0.36 (18)
C1—C6—C7—N1	−75.26 (14)	C13—O3—C12—O2	0.36 (18)
C5—C6—C7—N1	99.89 (14)	C13—O3—C12—C10	−178.13 (10)
C1—C6—C7—C10	47.66 (15)	C9—C10—C12—O2	10.07 (19)
C5—C6—C7—C10	−137.19 (13)	C7—C10—C12—O2	−167.88 (12)
C7—N1—C8—O1	−160.27 (11)	C9—C10—C12—O2	10.07 (19)
C7—N1—C8—N2	22.03 (16)	C7—C10—C12—O2	−167.88 (12)
C9—N2—C8—O1	−175.96 (11)	C9—C10—C12—O3	−171.52 (10)
C9—N2—C8—N1	1.82 (17)	C7—C10—C12—O3	10.53 (14)
C8—N2—C9—C10	−12.97 (18)	C12—O3—C13—C14	177.11 (13)

### Hydrogen-bond geometry (Å, °)

**Table:**

*D*—H···*A*	*D*—H	H···*A*	*D*···*A*	*D*—H···*A*
N1—H1···O2^i^	0.86	2.37	3.1773 (13)	156
N2—H2···O1^ii^	0.86	2.00	2.8568 (13)	178
C11—H11A···O1^iii^	0.96	2.58	3.1785 (16)	121
C11—H11C···O2	0.96	2.44	2.8379 (17)	105

Symmetry codes: (i) *x*−1, *y*, *z*; (ii) −*x*, −*y*+2, −*z*+1; (iii) *x*+1, *y*, *z*.

## References

[bb1] Atwal, K. S., Swanson, B. N., Unger, S. E., Floyd, D. M., Moreland, S., Hedberg, A. & O Reilly, B. C. (1991). *J. Med. Chem.* **34**, 806–811. 10.1021/jm00106a048 1995904

[bb2] Broughton, B. J., Chaplen, P., Knowles, P., Lunt, E., Marshall, S. M., Pain, D. L. & Wooldridge, K. R. H. (1975). *J. Med. Chem.* **18**, 1117–1122. 10.1021/jm00245a014 809582

[bb3] Bruker (2004). *APEX2* and *SAINT* Bruker AXS Inc., Madison Wisconsin, USA.

[bb4] Cremer, D. & Pople, J. A. (1975). *J. Am. Chem. Soc.* **97**, 1354–1358.

[bb5] Farrugia, L. J. (1997). *J. Appl. Cryst.* **30**, 565.

[bb6] Kappe, C. O., Fabian, W. M. F. & Semones, M. A. (1997). *Tetrahedron*, **53**, 2803–2816.

[bb7] Li, M., Guo, W.-S., Wen, L.-R. & Qi, W.-Y. (2005). *Acta Cryst.* E**61**, o531–o533.

[bb8] Nardelli, M. (1983). *Acta Cryst.* C**39**, 1141–1142.

[bb9] Overman, L. E., Michael, H., Rabinowitz, M. H. & Renhowe, P. A. (1995). *J. Am. Chem. Soc.* **117**, 2657–2658.

[bb10] Sheldrick, G. M. (2008). *Acta Cryst.* A**64**, 112–122. 10.1107/S0108767307043930 18156677

[bb11] Spek, A. L. (2003). *J. Appl. Cryst.* **36**, 7–13.

